# Hydroxygenkwanin Increases the Sensitivity of Liver Cancer Cells to Chemotherapy by Inhibiting DNA Damage Response in Mouse Xenograft Models

**DOI:** 10.3390/ijms22189766

**Published:** 2021-09-09

**Authors:** Chin-Chuan Chen, Chi-Yuan Chen, Shu-Fang Cheng, Tzong-Ming Shieh, Yann-Lii Leu, Wen-Yu Chuang, Kuang-Ting Liu, Shir-Hwa Ueng, Yin-Hwa Shih, Li-Fang Chou, Tong-Hong Wang

**Affiliations:** 1 Tissue Bank, Chang Gung Memorial Hospital, Taoyuan 33305, Taiwan; chinchuan@mail.cgu.edu.tw (C.-C.C.); d49417002@gmail.com (C.-Y.C.); ylleu@mail.cgu.edu.tw (Y.-L.L.); 2 Graduate Institute of Natural Products, Chang Gung University, Taoyuan 33303, Taiwan; wazai1023@gmail.com; 3 Graduate Institute of Health Industry Technology and Research Center for Industry of Human Ecology, College of Human Ecology, Chang Gung University of Science and Technology, Taoyuan 33303, Taiwan; 4 School of Dentistry, College of Dentistry, China Medical University, Taichung 40402, Taiwan; tmshieh@mail.cmu.edu.tw; 5 Department of Anatomic Pathology, Chang Gung Memorial Hospital, Taoyuan 33305, Taiwan; chuang.taiwan@gmail.com (W.-Y.C.); susie.ueng@gmail.com (S.-H.U.); 6 College of Medicine, Chang Gung University, Taoyuan 33303, Taiwan; 7 Department of Biomedical Sciences, National Chung Hsing University, Taichung 40227, Taiwan; shaintane@gmail.com; 8 Department of Pathology & Laboratory Medicine, Taoyuan Armed Forces General Hospital, Taoyuan 32551, Taiwan; 9 Department of Healthcare Administration, College of Medical and Health Science, Asia University, Taichung 41354, Taiwan; evashih@asia.edu.tw; 10 Kidney Research Center, Chang Gung Memorial Hospital, Taoyuan 33305, Taiwan; 11 Liver Research Center, Department of Hepato-Gastroenterology, Chang Gung Memorial Hospital, Taoyuan 33305, Taiwan

**Keywords:** hydroxygenkwanin, liver cancer, DNA damage response, RAD51, homologous recombination

## Abstract

Molecules involved in DNA damage response (DDR) are often overexpressed in cancer cells, resulting in poor responses to chemotherapy and radiotherapy. Although treatment efficacy can be improved with the concomitant use of DNA repair inhibitors, the accompanying side effects can compromise the quality of life of patients. Therefore, in this study, we identified a natural compound that could inhibit DDR, using the single-strand annealing yeast-cell analysis system, and explored its mechanisms of action and potential as a chemotherapy adjuvant in hepatocellular carcinoma (HCC) cell lines using comet assay, flow cytometry, Western blotting, immunofluorescence staining, and functional analyses. We developed a mouse model to verify the in vitro findings. We found that hydroxygenkwanin (HGK) inhibited the expression of RAD51 and progression of homologous recombination, thereby suppressing the ability of the HCC cell lines to repair DNA damage and enhancing their sensitivity to doxorubicin. HGK inhibited the phosphorylation of DNA damage checkpoint proteins, leading to apoptosis in the HCC cell lines. In the mouse xenograft model, HGK enhanced the sensitivity of liver cancer cells to doxorubicin without any physiological toxicity. Thus, HGK can inhibit DDR in liver cancer cells and mouse models, making it suitable for use as a chemotherapy adjuvant.

## 1. Introduction

According to the data published by the World Health Organization (WHO), approximately 700,000 people die from liver cancer each year [1]. Hepatocellular carcinoma (HCC) is the most common type of primary liver cancer. The primary treatment modalities for liver cancer include chemotherapy and radiotherapy. However, liver cancers are among the most difficult cancers to treat because of not only the genetic heterogeneity and high metastatic potential but also the abnormal expression of DNA repair proteins like MSH2, AXIN1, TP53, and RAD51 in advanced liver cancer cells, which leads to poor efficacy of chemotherapy and radiotherapy [2,3,4]. 

DNA damage response (DDR) is a key mechanism by which cells respond to genotoxic stress [5]. It can not only repair the endogenous DNA damage caused by abnormal DNA replication and reactive oxygen species generated during various metabolic processes, but also the exogenous DNA damage caused by factors such as radiation and toxic substances, thereby maintaining genome stability [6,7]. Upon detecting DNA damage, cells activate the DDR signaling cascade, which arrests cell cycle progression and activates appropriate DNA repair pathways involving multiple molecules, such as sensors, transducers, and effectors [8]. Cell cycle arrest allows ample time for DNA repair, thus preventing the daughter cells from inheriting the damaged DNA, thereby maintaining genome stability. Alternatively, cells activate the apoptosis, autophagy, or senescence pathway if the damage is beyond repair [9,10,11,12]. The repair mechanisms activated in human cells in response to various types of DNA damage include nucleotide excision repair, mismatch repair, base excision repair, and double-strand break repair (DSBR) [13]. There are two main pathways for DSBR, non-homologous end-joining and homologous recombination (HR). Different types of repair pathways are strictly regulated in the cells and activated based on the source of DNA damage.

DDR involves several key molecules, such as ataxia telangiectasia-mutated (ATM), ataxia telangiectasia- and Rad3-related (ATR), RAD51, RAD52, and poly (ADP-ribose) polymerases (PARP), which play crucial roles in different types of repair pathways [14]. Changes in the level of activity or reduced expression of these molecules can lead to defects in the DNA repair pathways and an increased probability of acquiring gene mutations, which in turn is closely associated with aging and diseases, including neurodegenerative disorders and cancer [15,16,17,18,19]. However, several cancers, including prostate, colorectal, pancreatic, breast, and non-small-cell lung cancers, have demonstrated an overexpression or activation of various DNA repair proteins, such as BRCA1/2, RAD51, ATM, MRE11, excision-repair cross-complementation (ERCC), and DNA repair protein complementing XP-C cells (XPC), which reduce the sensitivity of the tumors to chemotherapy and radiotherapy [20,21,22,23,24]. Therefore, a large number of anti-cancer drugs have been developed to target these DNA repair proteins [18,25]. Food and Drug Administration (FDA)-approved PARP inhibitors, such as olaparib, rucaparib, and niraparib, are used as single agents in the treatment of BRCA1/2-mutant ovarian cancer, as they trigger synthetic lethality [26]. Additionally, the preliminary results of the clinical trials on RAD51 inhibitors, such as B02, DIDS, and RI-1, suggest that they can improve the sensitivity of cancer cells to chemotherapeutic drugs when used as adjuvants [27,28,29,30].

Upon detection of DNA damage, cells not only initiate DNA repair, but also activate the DNA damage checkpoint proteins, ATM and ATR, which phosphorylate the downstream molecules such as p53, checkpoint kinase 1 (CHK1), and checkpoint kinase 2 (CHK2), and arrest the cell cycle [31]. Recent studies have shown that activation of ATM/ATR, besides regulating the cell cycle, could induce autophagy and help cancer cells survive; this makes them important targets for future anti-cancer therapies [32,33]. Several ongoing preclinical and clinical trials on ATM/ATR inhibitors have also confirmed that they can enhance the efficacy of chemotherapy and radiotherapy [34,35].

Although the efficacy of chemotherapy and radiotherapy can be improved by concomitantly administering DNA repair inhibitors, the accompanying physiological side effects can considerably compromise the quality of life of patients. Therefore, research is focused on the development of DNA repair inhibitors with reduced side effects and superior efficacy for the treatment of cancer.

Owing to the diverse pharmacophores, highly complex stereochemistry, and low physiological toxicity in most species, natural products have been an important source of lead compounds for the development of anti-cancer drugs. Several natural compounds and their derivatives, such as paclitaxel, curcumin, and camptothecin, have demonstrated good results in clinical trials for the treatment of melanoma and cancers of the breast, lung, colon and rectum, etc., leading to a considerable improvement in the patient survival [36,37]. Furthermore, multiple Chinese herbal extracts, including sulforaphane, resveratrol, and diallyl disulfide, inhibit DDR, making them suitable for use as chemotherapy or radiotherapy adjuvants in cancer treatment [38,39,40,41].

*Daphne genkwa* is a commonly used Chinese herbal medicine in Southeast Asian countries, that is known to exhibit anti-inflammatory, antibacterial, and anti-cancer effects [42,43]. Hydroxygenkwanin (HGK) is a flavonoid extracted from *Daphne genkwa*, which not only has anti-inflammatory properties, but also induces mitochondrial damage and DNA breaks in glioma cells, leading to cell cycle arrest and apoptosis [44,45]. In addition, our previous research has shown that by inhibiting the expression of the transcription factor, FOXM1, and Class I histone deacetylase (HDAC), HGK can inhibit the growth, migration, and invasion of liver cancer cells, thereby promoting cellular apoptosis [46,47]. In this study, we aimed to identify a natural compound that could inhibit DDR, using a single-strand annealing (SSA) yeast-cell analysis system. We found that HGK sensitized yeast cells to single DSBs, and explored the mechanism of action and clinical application potential of the HGK using HCC cell lines and mouse models. 

## 2. Results

### 2.1. HGK Inhibits DDR and Promotes Apoptosis in Yeast Cells

**Figure 1:**
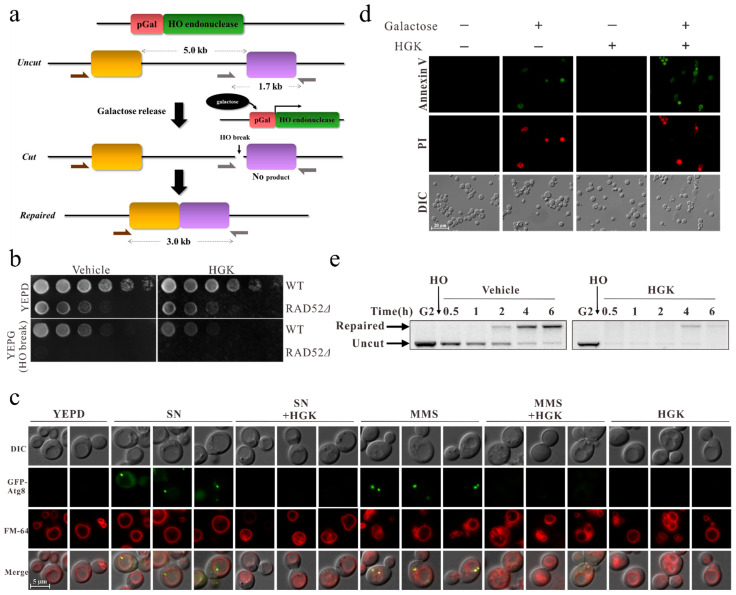
Hydroxygenkwanin (HGK) inhibits DDR in yeast cells and promotes apoptosis. (**a**) Schematic representation of the single-strand annealing (SSA) analysis system. Galactose-induced HO endonuclease results in the generation of specific double-strand DNA breaks in a defined location (HO break). The effect of HGK on double-strand break repair (DSBR) was analyzed by polymerase chain reaction (PCR) using the indicated primers. (**b**) Five-fold serial dilution analysis shows the sensitivity of the WT (YMV045) and RAD52∆ (YMV046) strains to HGK and double-strand DNA breaks. (**c**) The GFP-ATG8 yeast cells (RLY004 strain, transformed with a plasmid containing a green fluorescent protein fused with ATG8) were cultured in yeast extract peptone dextrose growth medium, nitrogen starvation (SN), MMS (0.1%), HGK (2 mM), SN + HGK, and MMS + HGK for 4 h. Samples were stained with FM4-64 and processed for fluorescence microscopy. DIC served as controls, and all scale bars represent 5 μm. (**d**) Yeast cells (YMV045) were treated with 2 mM HGK in the presence or absence of 2% galactose for 48 h, and cell apoptosis was determined by Annexin V/PI staining. (**e**) A double-strand DNA break was induced in YMV045 cells by galactose. The effect of HGK on DSBR was examined by PCR. MMS: methyl methanesulfonate; DIC: differential interference contrast microscopy.

To identify compounds that can inhibit DDR, the previously established SSA yeast-cell analysis system was adopted for screening (Figure 1a) [41]. The natural compounds used for screening were kindly provided from Professor Yann-Lii Leu. We found that HGK, a natural compound extracted from *Daphne genkwa*, inhibited the growth of yeast cells that endured galactose-induced DNA breaks (Figure 1b). In addition, it suppressed autophagy induced by DNA damage, thereby promoting apoptosis in yeast cells (Figure 1c,d). These results suggested that HGK can increase the sensitivity of yeast cells to DNA damage.

Therefore, to explore the effect of HGK on the DDR of yeast cells, PCR was performed. We observed that yeast cells could initiate DNA repair after the induction of DNA breaks. In cells treated with the vehicle control, most of the damaged DNA molecules were repaired within 4 h. However, in those treated with HGK, only 42% of the damaged DNA molecules were repaired in the same duration (Figure 1e). The immunoblotting results also showed that HGK inhibited the expression of SSA essential protein RAD52 following DNA damage in yeast (Figure S1). This indicates that HGK can inhibit DDR in yeast cells, leading to apoptosis of cells whose DNA cannot be repaired.

### 2.2. HGK Inhibits DDR of Liver Cancer Cell Lines

**Figure 2:**
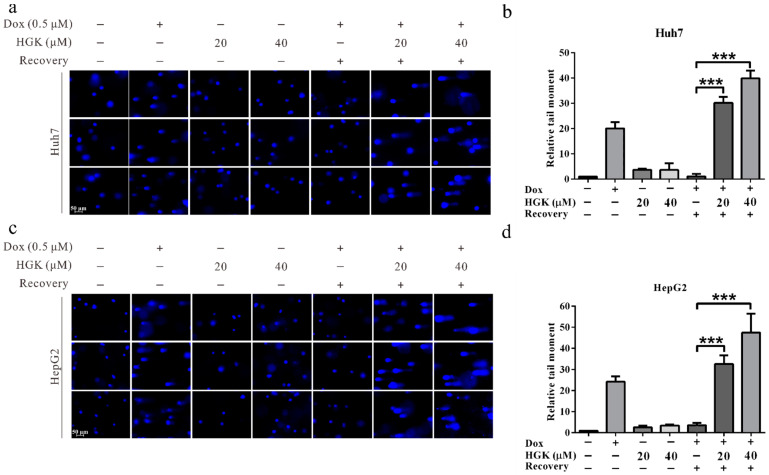
Hydroxygenkwanin (HGK) inhibits the DNA damage repair ability of hepatocellular carcinoma cell lines. (**a**,**c**) Huh7 and HepG2 cells were treated with 0.5 μM doxorubicin for 1 h, followed by treatment with different concentrations of HGK in doxorubicin-free medium for 4 h. The cells were harvested and subjected to alkaline denaturing comet assay to assess the DNA repair activity. (**b**,**d**) Quantitative DNA damage repair activity of the cells, *p* < 0.001 (***). All data were expressed as mean ± standard deviation values obtained from three independent experiments.

To understand whether HGK can exert an inhibitory effect on DDR in liver cancer cells, an alkaline denaturing comet assay was performed. In HCC cell lines, HGK alone could not induce DNA damage; therefore, doxorubicin was used to induce DNA damage prior to treatment with HGK. We observed that the rate of DNA damage repair in cells treated with HGK was significantly lower than that in the untreated control group. In addition, the inhibitory effect of HGK on DDR increased with an increase in the concentration of HGK. These findings suggest that HGK can inhibit DDR in liver cancer cell lines (Figure 2). 

### 2.3. HGK Inhibits HR Repair in Liver Cancer Cell Lines

**Figure 3:**
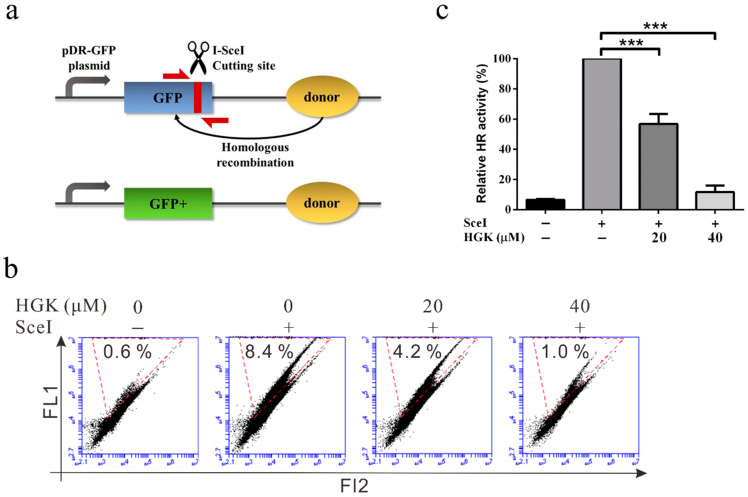
Hydroxygenkwanin (HGK) inhibits homologous recombination (HR) repair in hepatocellular carcinoma cell lines. (**a**) Schematic representation of the I-SceI-based HR assay. DR-GFP plasmid contains a truncated GFP-coding region that contains an I-SceI cutting site (blue). The I-SceI cleavage site can be repaired by HR using the downstream GFP donor template (yellow), resulting in GFP-positive cells (green). (**b**) Representative flow cytometry plots show the effect of HGK on HR activity in Huh7 cells. Huh7 cells were co-transfected with the DR-GFP vector and I-SceI expression vector with or without HGK treatment for 48 h to measure the HR-mediated repair capacity. The GFP-positive cells (area marked with red dashed line) were then detected by flow cytometry. FL1: green fluorescence; FL2: orange fluorescence. (**c**) Quantitative results for HR activity. *p* < 0.001 (***).

Previous studies have suggested that HR is the primary mechanism underlying DSBR in cancer cells [48]. To understand whether HGK can inhibit DDR by suppressing HR, the effect of HGK on the ability of the cells to perform HR was analyzed using a DR-GFP system and flow cytometry (Figure 3a). We observed that in HCC cell lines, the HR activity of the cells treated with 20 µM HGK was approximately 50% lower than that of those treated with the vehicle control (4.2% vs. 8.4%). In addition, when the concentration of HGK was increased to 40 µM, the HR activity was almost completely inhibited (1%). This suggests that HGK can inhibit the progression of HR in liver cancer cell lines (Figure 3b,c). 

The occurrence of HR is restricted to late S to G2 phases. To understand whether HGK affects cell cycle progression, flow cytometry analysis was performed. The results showed that compared with the vehicle control group, HGK treatment caused cell cycle arrest in the G2/M-phase. This suggests that HGK may inhibit HR and delay DNA repair, which in turn leads to cell cycle arrest (Figure S2).

### 2.4. HGK Inhibits HR Progression by Suppressing the Expression of RAD51

**Figure 4:**
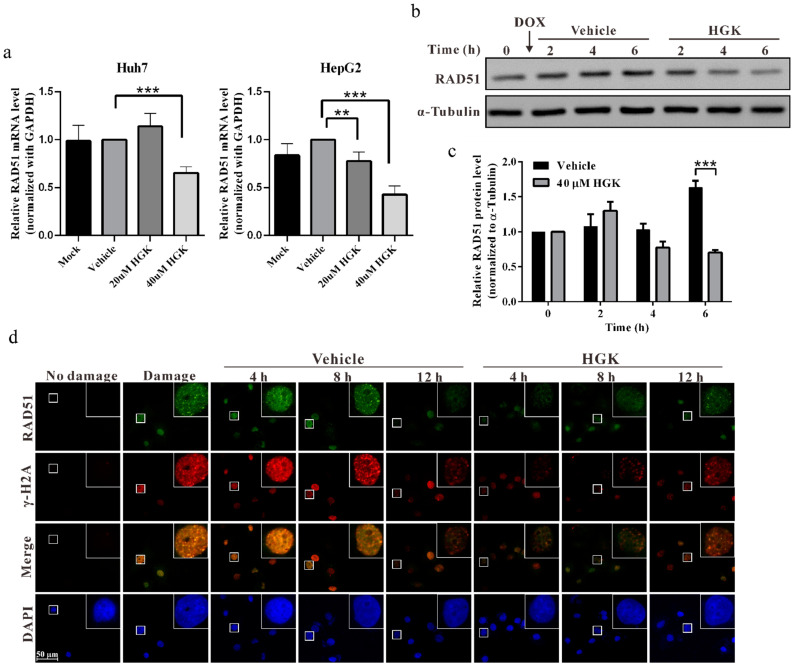
Hydroxygenkwanin (HGK) inhibits HR by suppressing the expression of RAD51. Huh7 and HepG2 cells were treated with different concentrations of HGK or vehicle control for 48 h, and the cells were harvested and subjected to (**a**) quantitative reverse transcription-polymerase chain reaction and (**b**) Western blotting analysis to detect the expression of RAD51. Glyceraldehyde 3-phosphate dehydrogenase and α-tubulin served as internal controls. (**c**) Quantitative results of the Western blotting analysis. Mock: cells treated with DMEM medium. Vehicle: cells treated with DMSO. *p* < 0.01 (**), *p* < 0.001 (***). All data were expressed as mean ± standard deviation values obtained from three independent experiments. (**d**) Huh7 cells were treated with 0.5 μM doxorubicin for 1 h to induce DNA damage, followed by recovery in a medium with or without 40 μM HGK. Cells were processed for immunofluorescence staining at various time points to detect the formation of RAD51 (green) and γH2AX (red) foci. Nuclei were stained with 4′,6-diamidino-2-phenylindole (blue).

RAD51 is a key protein that regulates HR [49]. To investigate whether HGK can inhibit the progression of HR by regulating RAD51, quantitative reverse transcription (RT)-PCR and Western blotting were performed to explore the effect of HGK on the expression of RAD51 mRNA and protein. We observed that the expression of RAD51 mRNA and protein in HCC cell lines treated with HGK was substantially lower than that in HCC cell lines treated with the vehicle control (Figure 4a,c). This indicates that HGK can inhibit the progression of HR by suppressing the expression of RAD51. To further understand the expression and localization of RAD51 during DDR, an immunofluorescence staining analysis was performed; additionally, to understand the status of DDR, the γ-H2AX foci were analyzed (Figure 4d). We observed that in cells treated with the vehicle control, the expression of RAD51 increased considerably following doxorubicin treatment. RAD51 was located at the same position as the γ-H2AX foci, indicating that RAD51 was recruited to the site of DNA damage. Subsequently, as DNA damage was gradually repaired, the expression of RAD51 and γ-H2AX foci decreased. In contrast, in cells treated with HGK, the expression of RAD51 and the γ-H2AX foci was significantly reduced. This suggests that HGK can inhibit the expression of RAD51 and DNA damage checkpoint proteins.

### 2.5. HGK Inhibits Phosphorylation of DNA Damage Checkpoint Proteins ATM/ATR

**Figure 5:**
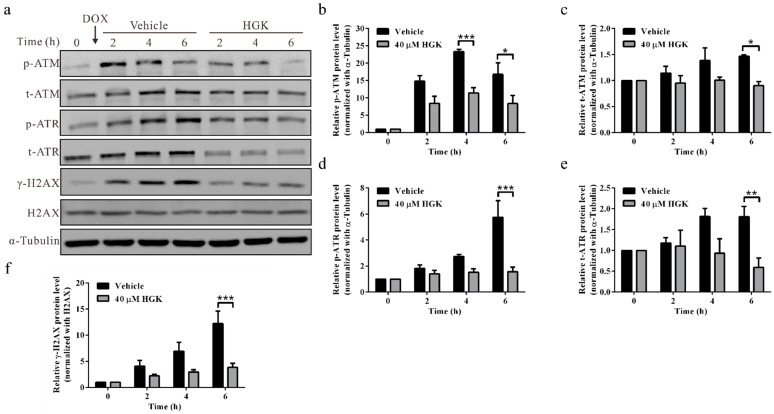
Hydroxygenkwanin (HGK) inhibits the activation of DNA damage checkpoint proteins, ataxia telangiectasia-mutated (ATM) and ataxia telangiectasia- and Rad3-related (ATR). (**a**) Huh7 cells were treated with 0.5 μM doxorubicin for 1 h, followed by treatment with 40 μM HGK in doxorubicin-free medium. Cells were harvested at indicated time points, and the activation of ATM and ATR was analyzed by Western blotting. α-tubulin served as an internal control. (**b**–**f**) Quantitative results for ATM and ATR activation. All experiments were performed in triplicate. p-ATM/ATR: phosphorylated ATM/ATR; t-ATM/ATR: total ATM/ATR. *p* < 0.05 (*), *p* < 0.01 (**), *p* < 0.001 (***).

Upon detection of double-strand DNA breaks, the DNA damage checkpoint proteins, ATM and ATR, undergo phosphorylation and activate the downstream CHK1 and CHK2 signaling pathways; this arrests the cell cycle progression, thereby allowing ample time for DNA repair [50]. In addition, ATM/ATR recruits DDR-related proteins to the site of DNA damage by phosphorylating H2AX. To elucidate the mechanism by which HGK inhibits DNA damage checkpoint signaling, a Western blotting analysis was performed to analyze the expression and phosphorylation of ATM and ATR. We observed that in cells treated with the vehicle control, ATM, ATR, and H2AX were phosphorylated in response to doxorubicin-induced DNA damage, suggesting that the DNA damage checkpoint proteins were conventionally activated. Contrarily, in the cells treated with HGK, the phosphorylation of ATM and ATR was considerably reduced, resulting in reduced phosphorylation of H2AX (Figure 5). These findings indicate that HGK can inhibit the phosphorylation of ATM and ATR in response to doxorubicin-induced DNA damage, thereby inhibiting DNA damage checkpoint signaling. Furthermore, by compromising the formation of γ-H2AX foci and the subsequent recruitment of DNA repair proteins, HGK can reduce the efficiency of DNA repair.

### 2.6. HGK Improves the Efficacy of Chemotherapy in HCC Cell Lines

**Figure 6:**
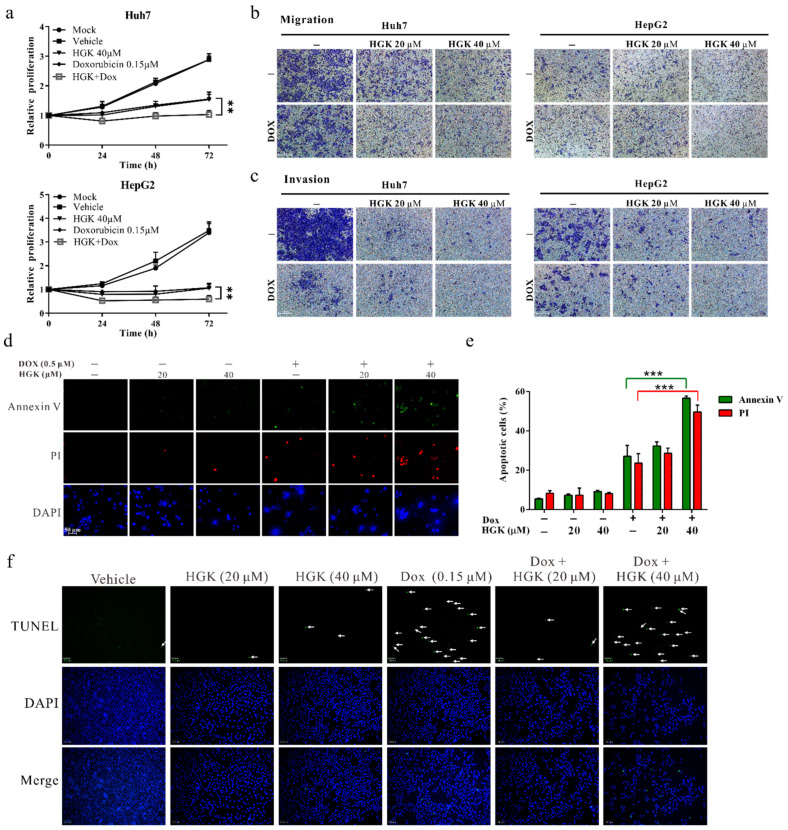
Hydroxygenkwanin (HGK) increases the sensitivity of hepatocellular carcinoma cell lines to doxorubicin. (**a**–**c**) The effect of HGK combined with doxorubicin on the proliferation, migration, and invasion of Huh7 and HepG2 cell lines. *p* < 0.01 (**), *p* < 0.001 (***). (**d**–**f**) The effect of HGK combined with doxorubicin on apoptosis in Huh7 cells was analyzed using Annexin V/PI staining and terminal deoxynucleotidyl transferase dUTP nick-end labeling (TUNEL) assay. Green punctate staining represents TUNEL-positive cells (white arrow). All experiments were performed in triplicates.

Most cancer cells respond poorly to chemotherapy and radiotherapy because of the abnormal expression and activation of DNA repair genes. To investigate the feasibility of using HGK as a chemotherapy adjuvant, HCC cell lines were treated with HGK and the chemotherapeutic drug, doxorubicin, either alone or in combination with each other. Following this, a functional analysis was performed to explore the effects of the two agents on cancer cells. Although both HGK and doxorubicin inhibited the growth, migration, and invasion of HCC cell lines when administered individually, their combination improved this inhibitory effect by up to 80%, thus demonstrating the ability of HGK to improve the efficacy of doxorubicin (Figure 6a–c). In addition, an apoptosis assay was performed to determine whether HGK can enhance the efficacy of chemotherapy in HCC cell lines. Results of the Annexin V/PI staining showed that in HCC cell lines, treatment with HGK alone did not significantly increase apoptosis, whereas treatment with doxorubicin led to apoptosis in approximately 21.22% of the cells. Moreover, treatment with HGK and doxorubicin combined led to a two-fold (44.62%) increase in apoptosis (Figure 6d,e). Similar results were observed in the terminal deoxynucleotidyl transferase dUTP nick-end labeling (TUNEL) assay (Figure 6f), indicating that HGK can increase the sensitivity of cancer cells to chemotherapeutic drugs.

### 2.7. HGK Improves the Efficacy of Doxorubicin in HCC Cell Lines by Inhibiting RAD51-Mediated DDR

**Figure 7:**
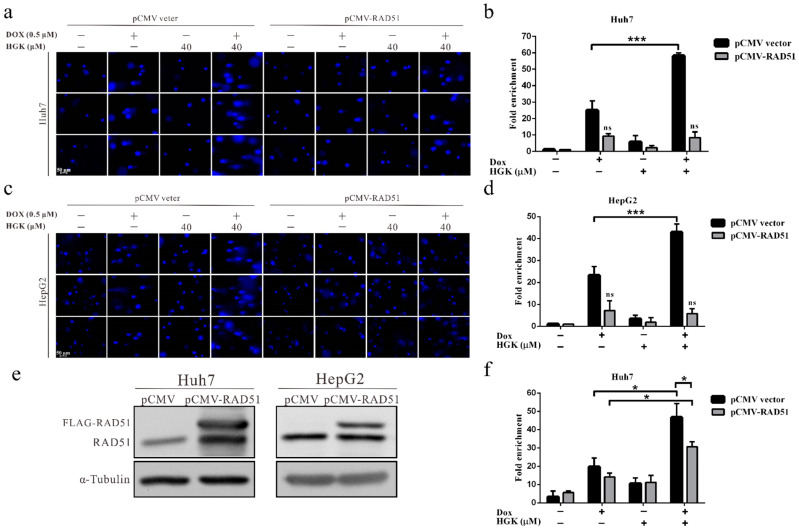
Hydroxygenkwanin (HGK) improves the efficacy of doxorubicin by inhibiting RAD51-mediated DDR in hepatocellular carcinoma cell lines. (**a**,**c**) Huh7 and HepG2 cells were transfected with pCMV-FLAG-RAD51 plasmid or vector for 48 h, followed by treatment with 40 μM HGK and 0.5 μM doxorubicin for 2 h. The cells were harvested and subjected to alkaline denaturing comet assay to assess the DNA repair activity. The results show that the overexpression of the RAD51 protein attenuates the inhibition of DNA repair by HGK in Huh7 and HepG2 cells. (**b**,**d**) Quantitative results of the cell repair activity. Data were expressed as mean ± standard deviation of three independent experiments. *p* < 0.05 (*), *p* < 0.001 (***). (**e**) Western blotting analysis shows the expression of FLAG-tagged RAD51 and endogenous RAD51 in transfected cells. (**f**) Trypan blue staining shows that HGK enhances doxorubicin-induced apoptosis, whereas RAD51 overexpression significantly decreases the apoptosis ratio.

To verify that HGK inhibited DDR in HCC cell lines by suppressing the expression of RAD51, thereby enhancing the efficacy of doxorubicin, a rescue assay was performed. The results of the comet assay showed that the HCC cell lines treated with HGK demonstrated a substantially reduced rate of DNA repair. However, overexpression of the RAD51 protein following treatment with HGK, led to an almost complete abrogation of the inhibitory effect of HGK on DNA repair (Figure 7a–e). In addition, the apoptosis assay showed that when RAD51 was overexpressed, the additive effect of HGK and doxorubicin was reduced by approximately 40% (Figure 7f). These findings indicate that HGK can inhibit DDR in HCC cell lines by suppressing the expression of RAD51, thereby enhancing the efficacy of doxorubicin.

### 2.8. HGK Increases the Sensitivity of Liver Cancer Cells to Doxorubicin In Vivo

**Figure 8:**
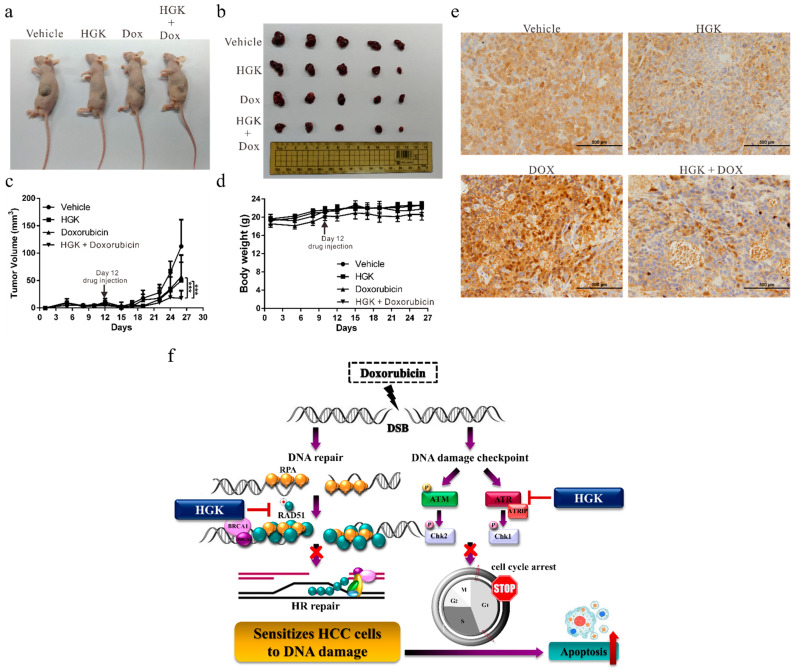
Hydroxygenkwanin (HGK) improves the sensitivity of liver cancer cells to doxorubicin in vivo. (**a**,**b**) Huh7 cells (5 × 10^6^) were injected into the flanks of BALB/c mice (n = 5 in each group). All tumors were staged for 12 days before drug treatment was initiated. The mice were intraperitoneally injected thrice weekly with HGK, doxorubicin, or an equal volume of dimethyl sulfoxide, which served as the control. Representative images show the tumor xenografts at 4 weeks post-implantation. (**c**) Tumor volume was measured every 3 days with calipers, using the following formula: volume = length × width^2^ × 0.5. The bars indicate standard deviation. *p* < 0.001 (***). (**d**) Body weight was calculated every 3 days. (**e**) Immunohistochemical staining showed a reduced expression of RAD51 in HGK-treated mice xenograft tumors. Magnification: 400×, scale bar: 500 μm. (**f**) A schematic representation summarizing the mechanism by which HGK suppresses DNA repair.

To explore the potential for clinical application of HGK, a mouse xenograft model was established to verify the in vitro findings. The Huh7 cells were injected into the flanks of the mice, following which, HGK and doxorubicin, either alone or in combination with each other, were regularly injected intraperitoneally. We observed that while the administration of HGK and doxorubicin alone inhibited tumor growth in mice, their joint administration demonstrated a considerable improvement in the inhibitory effect. This indicated an additive effect between HGK and doxorubicin (Figure 8a–c). Moreover, compared to the control mice, mice treated with HGK alone did not show any significant weight loss (Figure 8d) or reduction in activity, suggesting that HGK caused no apparent physiological toxicity.

Further, in order to confirm the mechanism by which HGK regulates DNA repair, immunohistochemical staining of the tumors was performed to assess the expression of RAD51. We observed that the expression of RAD51 was significantly lower in the mice treated with HGK than in the untreated control group (Figure 8e). These findings were consistent with those obtained from in vitro studies, suggesting that HGK can inhibit the expression of RAD51, resulting in apoptosis in liver cancer cells because of the lack of an appropriate response to DNA damage (Figure 8f).

## 3. Discussion

DDR plays an important role in maintaining genome stability and regulating cell survival by repairing the DNA damage caused by exogenous and endogenous factors. The overexpression or activation of DNA repair proteins is commonly observed in cancer cells and this reduces their sensitivity to chemotherapy and radiotherapy. Therefore, DNA repair inhibitors are used as chemotherapy or radiotherapy adjuvants in cancer treatment. In this study, we found that HGK, a compound extracted from *Daphne genkwa*, could inhibit DDR in HCC cell lines. In addition to suppressing the progression of HR by inhibiting the expression of RAD51, HGK could also inhibit the phosphorylation of the DNA damage checkpoint proteins, ATM and ATR, resulting in apoptosis in the HCC cell lines induced by failure to repair the DNA damage. To the best of our knowledge, ours is the first study to verify the inhibitory effect of HGK on DDR in xenograft mouse models. We also found that the administration of HGK significantly improved the efficacy of the chemotherapeutic drug, doxorubicin, in both in vitro and in vivo studies.

RAD51 is overexpressed in various cancers, including malignant gliomas and cancers of the liver, ovary, breast, lung, pancreas, colon, and rectum [51]. In addition, its expression shows a significant negative correlation with disease prognosis [52]. Previous studies on lung cancer have shown that the inhibition of RAD51 expression can reduce the HR repair ability of cancer cells and increase their sensitivity to chemotherapy and radiotherapy [53,54]. This observation is consistent with the results of our study. We found that HGK could improve the sensitivity of cancer cells to doxorubicin by inhibiting the expression of RAD51. Moreover, previous studies suggest that the p53 protein can regulate the expression of RAD51 by binding to the p53 response element on the RAD51 promoter and inhibiting the transcription of the RAD51 gene [55]. Our previous study has shown that HGK can improve the binding ability of p53 to the target gene promoter by stimulating the acetylation of p53 [46]. Therefore, we hypothesized that the acetylation of p53 due to HGK can promote the binding of p53 to the RAD51 promoter, thereby negatively regulating the expression of RAD51.

To verify whether HGK suppressed DNA repair and improved the efficacy of doxorubicin by inhibiting RAD51, a rescue assay was performed. We found that RAD51 overexpression in cells treated with both HGK and doxorubicin led to an almost complete abrogation of the inhibitory effect of HGK on DDR; moreover, RAD51 overexpression reduced the effect of HGK and doxorubicin by about 40% (Figure 7). This suggests that HGK might also regulate other tumor suppressor pathways to exert an additive effect on the anti-cancer effect of doxorubicin. Our previous studies demonstrated that HGK can inhibit the expression of FOXM1 and Class I HDACs to suppress the growth of cancer cells [46,47]. This suggests that even in the absence of the inhibitory effect of HGK on DNA repair, it can enhance the efficacy of doxorubicin by regulating other tumor suppressor signaling pathways.

Upon detection of DNA damage, cells not only initiate DNA repair, but also activate the DNA damage checkpoint to promote cell cycle arrest. ATM and ATR are key DNA damage checkpoint proteins. DNA damage triggers the biding of the Mre11-Rad50-Nbs1 complex and replication protein A to the DNA at the site of damage and induces the phosphorylation of ATM and ATR; following this, ATM and ATR regulate the activity of the protein cell division cycles 25 A and p53, to arrest cell cycle progression by activating the downstream CHK1 and CHK2 pathways [56]. Our experiments on HCC cell lines revealed that HGK can inhibit not only the HR repair pathway, but also the phosphorylation of ATM and ATR, thus, inhibiting the DNA damage checkpoint signaling. As a result, cells with damaged DNA can continue to divide and replicate without the ability to fully repair the damage, eventually leading to chromosomal abnormalities and apoptosis in the daughter cells.

There are multiple mechanisms of DNA repair in mammalian cells, and the type of repair mechanism corresponds to the type of DNA damage induced by the chemotherapeutic drugs. HR is the primary repair mechanism employed by cancer cells upon the induction of double-strand DNA breaks. As HGK inhibits HR, it can enhance the efficacy of the chemotherapeutic drugs such as doxorubicin, etoposide, and gemcitabine, which induce double-strand DNA breaks. The results from our study also suggest that the combined administration of HGK and doxorubicin exerts an additive effect on tumor suppression.

Autophagy is an important mechanism by which cells produce energy in response to stress. In response to starvation, autophagy can break down cellular proteins and non-essential components to generate energy and assist in cell survival. In addition, autophagy can help clear the damaged and aging organelles to maintain physiological stability and protect against viral and bacterial invasion [57]. Abnormal autophagy has been associated with cancer and other diseases [58]. Several studies have demonstrated that autophagy helps cancer cells overcome nutritional limitations and promotes cell growth [59,60,61]. Therefore, the inhibition of autophagy in cancer cells appears to be an appropriate treatment strategy. Our results suggest that HGK can effectively inhibit autophagy in yeast cells, even in the presence of a nutritional deficiency and DNA damage, an observation that has not been reported before. Although the potential inhibitory effect of HGK on autophagy in liver cancer cells and the underlying regulatory mechanisms requires further investigation, our study shows that in addition to inhibiting DNA repair, HGK also demonstrates other mechanisms of tumor suppression.

In conclusion, HGK can inhibit DDR both in vitro and in vivo, without significant physiological toxicity, thus making it suitable for use as a chemotherapy adjuvant. 

## 4. Materials and Methods

### 4.1. Yeast Strains, Cell Lines, Antibodies, Plasmids, and Drugs

All yeast strain genotypes used in this study are listed in Table 1. The HCC cell lines, Huh7 and HepG2, purchased from the American Type Culture Collection (Manassas, VA, USA) were cultured in Dulbecco’s Modified Eagle Medium (DMEM) containing 10% fetal bovine serum (FBS) and incubated at 37 °C with 5% CO_2_. Polyclonal antibodies against RAD51, γH2AX, ATM, ATR, phospho-ATM, phospho-ATR, α-tubulin, and β-actin were purchased from Cell Signaling Technology (Beverly, MA, USA) and Genetex (Irvine, CA, USA). Secondary antibodies were purchased from Proteintech (Rosemont, IL, USA) and Santa Cruz Biotechnology (Santa Cruz, CA, USA). The pCMV-FLAG-RAD51 plasmid, DR-GFP vector, and I-SceI expression vector (for the HR assay) were a kind gift from Professor Chin-Chuan Chen. The compounds used for screening were synthesized by Professor Yann-Lii Leu from the Traditional Chinese Medicine Research Center of Chang Gung University. HGK powder (purity > 98%, as measured by high-performance liquid chromatography) was purchased from Shanghai BS Bio-Tech Co., Ltd. (Shanghai, China). DOX was purchased from Sigma-Aldrich (St. Louis, MO, USA). HGK was dissolved in dimethyl sulfoxide (DMSO) to obtain a stock concentration of 10 mM, which was then stored at −20 °C before use. DMSO was used as the vehicle control. 

### 4.2. HO Induction

Yeast strains were cultured for 12 h in the yeast extract peptone (YEP) medium containing lactic acid (YEPL). DSB was induced by adding 2% galactose, and samples were then collected for DNA cutting and repair analysis.

### 4.3. DNA Damage Sensitivity Plate Assay

Yeast cells were diluted in 5-fold increments and plated onto yeast extract peptone dextrose (YEPD) plates, YEP medium contains 2% galactose, or the indicated concentration of DNA-damage drug and compound. The cells were incubated at 30 °C for 2 d and photographed to record colony formation. 

### 4.4. DNA Cutting and Repair Analysis

DNA cutting and repair of the HO site in SSA yeast strains was performed using three primers (SSA1: CCGCTGAACATACCACGTTG; SSA2: CACTTCCAGATGAGGCGCTG; and SSA3: TGAACTCTGGTGTCTTTTAG). PCR amplification before a DSB yielded a 1.7-kb product, and analysis following repair by SSA yielded a 3.0-kb product. Primers corresponding to *RAD3* were included in the multiplex PCR as an internal control (RAD3A: GATAAGATTGCGACAAAAGAGGATA; RAD3D: GTGGGACGAGACGTTTAGATAGTAA).

### 4.5. Fluorescence Microscopy

Yeast samples were collected for apoptosis and autophagy assays and analyzed using a fluorescence microscope (Nikon Eclipse Ni-U; Nikon, Tokyo, Japan) equipped with 100× oil immersion objectives. Images were acquired with a DS-U3 CCD camera and controlled using NIS-Element BR 4.0 software (Nikon, Tokyo, Japan). In the apoptosis assay, samples were stained with Annexin V-FITC and PI using the Apoptosis Detection Kit (ACE Biolabs Co., Ltd., Taoyuan, Taiwan). For the autophagy assay, samples were incubated in YEPD containing 5 mg/mL FM4-64 at 30 °C in the dark for 30 min, and further treated with the indicated concentration of DNA-damage drug and compound.

### 4.6. Real-Time RT-PCR

Total RNA was extracted from cells using the TOOLSmart RNA extractor (BIOTOOLS Co., Ltd., Taipei, Taiwan) and RNeasy mini kit (QIAGEN, Gaithersburg, MD, USA) according to the manufacturer’s instructions. Complementary DNA (cDNA) was synthesized from total RNA using ToolScript MMLV RT Kit (BIOTOOLS Co., Ltd., Taipei, Taiwan). Quantitative real-time PCR was performed using a TaqMan Gene Expression Kit (Applied Biosystems, Foster City, CA, USA) and TOOLS 2x SYBR qPCR Mix (BIOTOOLS Co., Ltd., Taiwan) on an ABI StepOnePlus™ System (Applied Biosystems). Glyceraldehyde 3-phosphate dehydrogenase was used as an internal control. 

### 4.7. Western Blotting Analysis

Western blotting was performed as previously described [47]. Briefly, the Huh7 and HepG2 cells were treated with various concentrations of HGK or dimethyl sulfoxide (DMSO) for 48 h, followed by lysis in RIPA buffer (BIOTOOLS Co., Ltd., Taipei, Taiwan) containing protease inhibitors. Cell lysates (30 µg protein) were then subjected to Western blotting analysis using α-tubulin or β-actin as loading controls. Immunoreactive bands were visualized using the ECL system (NEN Life Science Products, Boston, MA, USA), and contents of each band were quantified using the ImageQuant 5.2 software (GE Healthcare, Piscataway, NJ, USA).

### 4.8. Cell Proliferation Assay

A total of 5 × 10^3^ cells were seeded in 96-well plates and treated with various concentrations of HGK for 24, 48, and 72 h. Cell proliferation was measured using the 3-[4,5-dimethylthiazol-2-yl]-2,5-diphenyl tetrazolium bromide (MTT) assay according to the manufacturer’s instructions. 

### 4.9. Cell Migration and Invasion Assays

The migration and invasion abilities of the Huh7 and HepG2 cells were assessed using a transwell chamber (Millipore; Merck KGaA, Darmstadt, Germany), as described previously [46]. For the migration assay, a total of 4 × 10^4^ cells in 100 µL serum-free DMEM with/without HGK were seeded in the upper well. The lower chambers were filled with 500 μL of complete medium (DMEM supplemented with 10% FBS). The chambers were incubated in a humidified 5% CO_2_ incubator at 37 °C for 24 h. The cells were fixed with methanol and stained with 1% crystal violet. The stained cells were visualized using a light microscope (Olympus BX-53, Tokyo, Japan) and ImagePro 6.2 software (Media Cybernetics Inc., Bethesda, MD, USA), and the number of migrated cells was counted manually in five random fields at 100× magnification. For the invasion assay, the membrane was coated with 30 mg/cm^2^ of Matrigel (ECM gel, Sigma-Aldrich, St. Louis, MO, USA) to form a matrix barrier. The rest of the procedure for the invasion assay was the same as that for the migration assay, except that the permeation time for the cells was 48 h.

### 4.10. Apoptosis Assay

Apoptosis status of the Huh7 cells was determined using the DeadEndTM Fluorometric TUNEL Assay Kit (Promega, Madison, WI, USA) and Annexin V-FITC/PI Apoptosis Detection Kit (Thermo Scientific, Waltham, MA, USA) according to the manufacturer’s instructions. The Huh7 cells were grown on chamber slides and treated with different concentrations of HGK and doxorubicin for 48 h. The cells were fixed with 4% paraformaldehyde for 15 min at room temperature, following which they were subjected to a TUNEL assay and Annexin V/PI staining. Apoptotic cells were visualized using a fluorescence microscope (×100 magnification) and analyzed from five different fields of vision for each experiment.

### 4.11. Comet Assay

A total of 4 × 10^4^ cells were seeded in 24-well plates and treated with doxorubicin (0.5 µM) for 1 h. Doxorubicin was then washed off using phosphate-buffered saline (PBS), and the cells were treated with various concentrations of HGK or DMSO in a doxorubicin-free medium for 4 h. Cells were harvested by trypsinization, washed in ice-cold PBS, and subjected to the comet assay as described previously [62]. Comet images were obtained using a fluorescence microscope (Nikon ECLIPSE Ni-U plus; Nikon, Tokyo, Japan), and the tail moment was calculated using the OpenComet software.

### 4.12. HR Assay

The Huh7 cells were transfected with both the DR-GFP vector and I-SceI expression vector to determine the HR-mediated repair capacity after 48 h. Green fluorescent protein-positive cells were then detected using a Becton-Dickinson FACSCalibur flow cytometer.

### 4.13. Cell-Cycle Analysis

Cells were trypsinized, washed twice with cold PBS, and fixed in 70% ethanol overnight. After incubating in 1 mL of propidium iodide staining solution (0.1% Triton X-100, 100 μg/mL DNase-free RNase A, 20 μg/mL propidium iodide) for 1 h at room temperature. Cell cycle distribution was analyzed by flow cytometry (Beckman Coulter Epics Elite; Beckman, Inc., Fullerton, CA, USA).

### 4.14. Animal Experiments

Twenty male, 6-week-old BALB/c mice (National Laboratory Animal Center, Taipei, Taiwan) were subcutaneously injected with 5 × 10^6^ Huh7 cells in the left and right flank regions. The tumors were staged for 12 days before the initiation of drug treatment. Subsequently, the mice with tumors were intraperitoneally injected with 100 µL of HGK (at a dose of 1.5 mg/kg of body weight), doxorubicin (at a dose of 4 mg/kg of body weight), or an equal volume of DMSO, which served as a control, thrice weekly. Tumor volume was measured three times a week using digital calipers, and 15 days after drug administration, the mice were euthanized, and the tumors were subjected to immunohistochemical staining to analyze the expression of RAD51. All animal experiments were approved by the Institutional Animal Care and Use Committee (IACUC) of the Chang Gung Memorial Hospital (IACUC approval no.: 2018031301, approval date: 19 June 2018).

### 4.15. Immunohistochemistry

The tumors were fixed in 4% paraformaldehyde for 24 h, dehydrated, and embedded in paraffin. Paraffin blocks were sectioned to 2 μm thickness and mounted onto glass slides. The tissue sections were deparaffinized, and the expression of RAD51 in the tissues was measured as described previously [62].

### 4.16. Statistical Analysis

Statistical analysis was performed using the Statistical Package for the Social Sciences (SPSS) 16.0 (SPSS Inc., Chicago, IL, USA), and Microsoft Excel 2007 (Microsoft Inc., Redmond, WA, USA), and the values were expressed as mean ± standard error of the mean. All statistical tests were two-sided, and *p*-values of significance were established at <0.05 (*), <0.01 (**), or <0.001 (***).

## Figures and Tables

**Table 1:** Genotypes of yeast strains used in this study.

Strain	Genotype	Source
YMV045	*ho hml∆::ADE1 mata∆::hisG hmr∆::ade1 leu2::leu2 (Asp-718-SalI)-URA3-pBR322-HOcs ade3::GAL::HO ade1 lys5 ura3-52 trp1 (trp1::hisG)*	James Haber
YMV046	*ho hml∆::ADE1 mata∆::hisG hmr∆::ADE1 leu2:HOcs ade3::GAL::HO ade1 lys5 ura3-52 trp1 (trp1::hisG) rad52∆::HPH (hygro)*	James Haber
RLY004	BY4741-atg8 transform with PRS416 GFP-Atg8 in URA drop media	This study

## Data Availability

The data presented in this study is contained within the article.

## Supplementary Materials

The following are available online at https://www.mdpi.com/article/10.3390/ijms22189766/s1.
