# Supplementary material for: Hydroxygenkwanin Increases the Sensitivity of Liver Cancer Cells to Chemotherapy by Inhibiting DNA Damage Response in Mouse Xenograft Models
Source: Int J Mol Sci. 2021 Sep 9;22(18):9766. doi: 10.3390/ijms22189766 (PMC8471855; doi:10.3390/ijms22189766)
Supplement: Supplementary file 1 [file ijms-22-09766-s001.zip › ijms-1347683-supplementary.pdf]

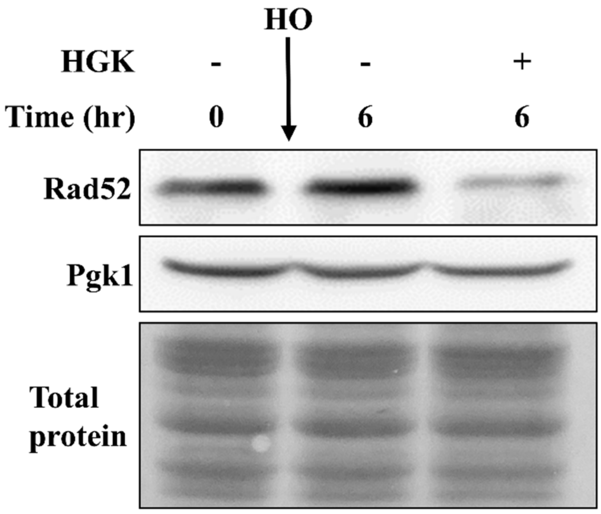

Figure S1. HGK inhibited the expression of RAD52 following DNA damage in yeast. The yeast cells (YMV045) were cultured in YEPL for 12 h, and HO endonuclease was induced by the addition of galactose at time 0 to generate a DSB. After 30 min, the cultures were divided equally and treated with or without 2 mM HGK. The immunoblots show the protein levels of Rad52 at the indicated time points. Pgk1 served as an internal control.

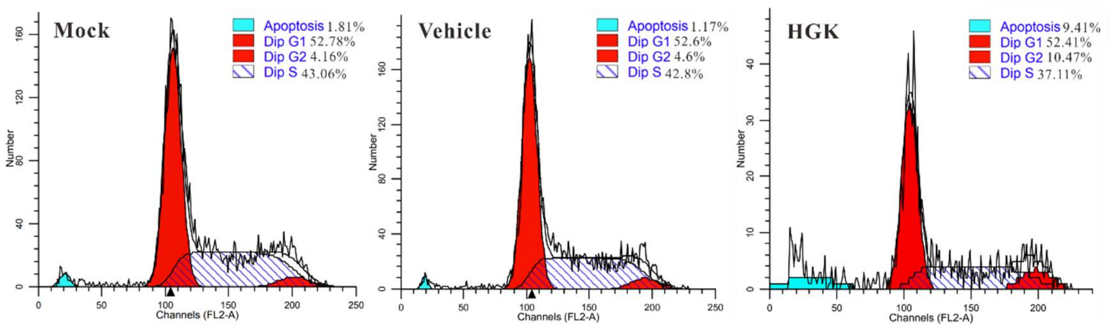

Figure S2. Effect of HGK on the cell cycle progression in Huh7 cells. Cells were treated with or without 40μM HGK for 24 h. The cell cycle distribution was analyzed by flow cytometry.
